# Radiomics-based machine learning model to phenotype hip involvement in ankylosing spondylitis: a pilot study

**DOI:** 10.3389/fimmu.2024.1413560

**Published:** 2024-08-29

**Authors:** Zhengyuan Hu, Yan Wang, Xiaojian Ji, Bo Xu, Yan Li, Jie Zhang, Xingkang Liu, Kunpeng Li, Jianglin Zhang, Jian Zhu, Xin Lou, Feng Huang

**Affiliations:** ^1^ Department of Rheumatology and Immunology, The First Medical Center, Chinese PLA General Hospital, Beijing, China; ^2^ Department of Radiology, The First Medical Center, Chinese PLA General Hospital, Beijing, China; ^3^ Basic Research Center for Medical Science, Academy of Medical Science, Zhengzhou University, Zhengzhou, Henan, China

**Keywords:** radiomics, spondylitis, ankylosing, hip involvement, machine learning, magnetic resonance imaging

## Abstract

**Objectives:**

Hip involvement is an important reason of disability in patients with ankylosing spondylitis (AS). Unveiling the potential phenotype of hip involvement in AS remains an unmet need to understand its biological mechanisms and improve clinical decision-making. Radiomics, a promising quantitative image analysis method that had been successfully used to describe the phenotype of a wide variety of diseases, while it was less reported in AS. The objective of this study was to investigate the feasibility of radiomics-based approach to profile hip involvement in AS.

**Methods:**

A total of 167 patients with AS was included. Radiomic features were extracted from pelvis MRI after image preprocessing and feature engineering. Then, we performed unsupervised machine learning method to derive radiomics-based phenotypes. The validation and interpretation of derived phenotypes were conducted from the perspectives of clinical backgrounds and MRI characteristics. The association between derived phenotypes and radiographic outcomes was evaluated by multivariable analysis.

**Results:**

1321 robust radiomic features were extracted and four biologically distinct phenotypes were derived. According to patient clinical backgrounds, phenotype I (38, 22.8%) and II (34, 20.4%) were labelled as high-risk while phenotype III (24, 14.4%) and IV (71, 42.5%) were at low risk for hip involvement. Consistently, the high-risk phenotypes were associated with higher prevalence of MRI-detected lesion than the low-risk. Moreover, phenotype I had significant acute inflammation signs than phenotype II, while phenotype IV was enthesitis-predominant. Importantly, the derived phenotypes were highly predictive of radiographic outcomes of patients, as the high-risk phenotypes were 3 times more likely to have radiological hip lesion than the low-risk [27 (58.7%) vs 16 (28.6%); adjusted odds ratio (OR) 2.95 (95% CI 1.10, 7.92)].

**Conclusion:**

We confirmed for the first time, the clinical actionability of profiling hip involvement in AS by radiomics method. Four distinct phenotypes of hip involvement in AS were identified and importantly, the high-risk phenotypes could predict structural damage of hip involvement in AS.

## Introduction

Ankylosing spondylitis (AS) is a chronic inflammatory disease that primarily involves the spine, sacroiliac joints and peripheral joints, which could potentially lead to significant morbidity and disability (1). Hip involvement is a prevalent manifestation and an important cause of disability in AS. It is also associated with spine damage, function impairment, increased disease burden and poor prognosis in AS (2, 3). Magnetic resonance image (MRI) can detect early hip lesion in AS and plays an important role in the diagnosis of hip involvement in AS (4). However, MRI-detected hip lesions like joint effusion, subchondral bone marrow edema (BME) were not AS-specific, they could also appear in a wide spectrum of clinical entities such as osteoarthritis, stress injury, femoral head avascular necrosis, joint infection and inflammatory disorders (5, 6). Moreover, it is prone to overestimate the prevalence of hip involvement in AS if we only rely on the present of abnormal MRI lesions (7) and the gold-standard MRI definition of hip involvement in AS is still lacking. Therefore, a new method that accurately predicts hip involvement in AS is urgently needed.

Radiomics has gained increasing attention over the last decade as a promising quantitative image analysis method that had been successfully used in patient phenotyping and prediction of treatment response in a wide variety of diseases (8, 9). Generally, radiomic features were firstly extracted from regions of interest (ROIs) in routine images like CT or MRI. Then, the radiomic features containing crucial information about disease were progressed by artificial intelligent techniques like machine learning (ML) or deep learning methods. Radiomics was initiated in oncology studies and extended to musculoskeletal diseases in the last few years (10). Moreover, ML-based deciphering of complex diseases, such as sepsis, heart failure, ARDS and COVID-19 (11–14), had successfully identified biologically distinct phenotypes and facilitated the understanding of their biological mechanisms. Therefore, we hypothesized that radiomics is a promising method in profiling of hip involvement in AS. We did this pilot study to evaluate the clinical actionability of using radiomics data to phenotype AS patients with symptomatic hip involvement and predict structural damage of hip joint in AS.

## Materials and methods

We retrospectively investigated AS patients with hip joint pain and who underwent pelvis MRI exams since January 2019 to September 2022, at the First Medical Center of the Chinese People’s Liberation Army (PLA) General Hospital, a tertiary referral center in Beijing. All enrolled patients met the following criteria: they were diagnosed with AS according to the 1984 modified New York criteria (15) and whose MRI imaging fulfilled the quality criteria for reading. Patients with other comorbidities that potentially result in hip joint pain were excluded. Socio-demographic data, type of previous anti-inflammatory medication (non-steroidal anti-inflammatory drugs (NSAIDs) and tumor necrosis factor inhibitors (TNFi)) and clinical assessments were obtained from medical records. Clinical assessments included age at onset, disease duration, peripheral arthritis history, serum inflammatory markers level (C-reactive protein (CRP) and erythrocyte sedimentation rate (ESR)) and HLA-B27 status. Furthermore, X-rays of anterior–posterior pelvis were collected and the severity of structure damage of hip joint was assessed by the Bath ankylosing spondylitis radiology hip index (BASRI-hip) (16). Research ethics approval was granted by the Ethical Committee of the Chinese PLA General Hospital (S2023-375-01) and informed consent was waived due to the retrospective nature of the study. Our works were conducted in accordance with the Declaration of Helsinki.

### MRI image acquisition and preprocessing

**Figure 1:**
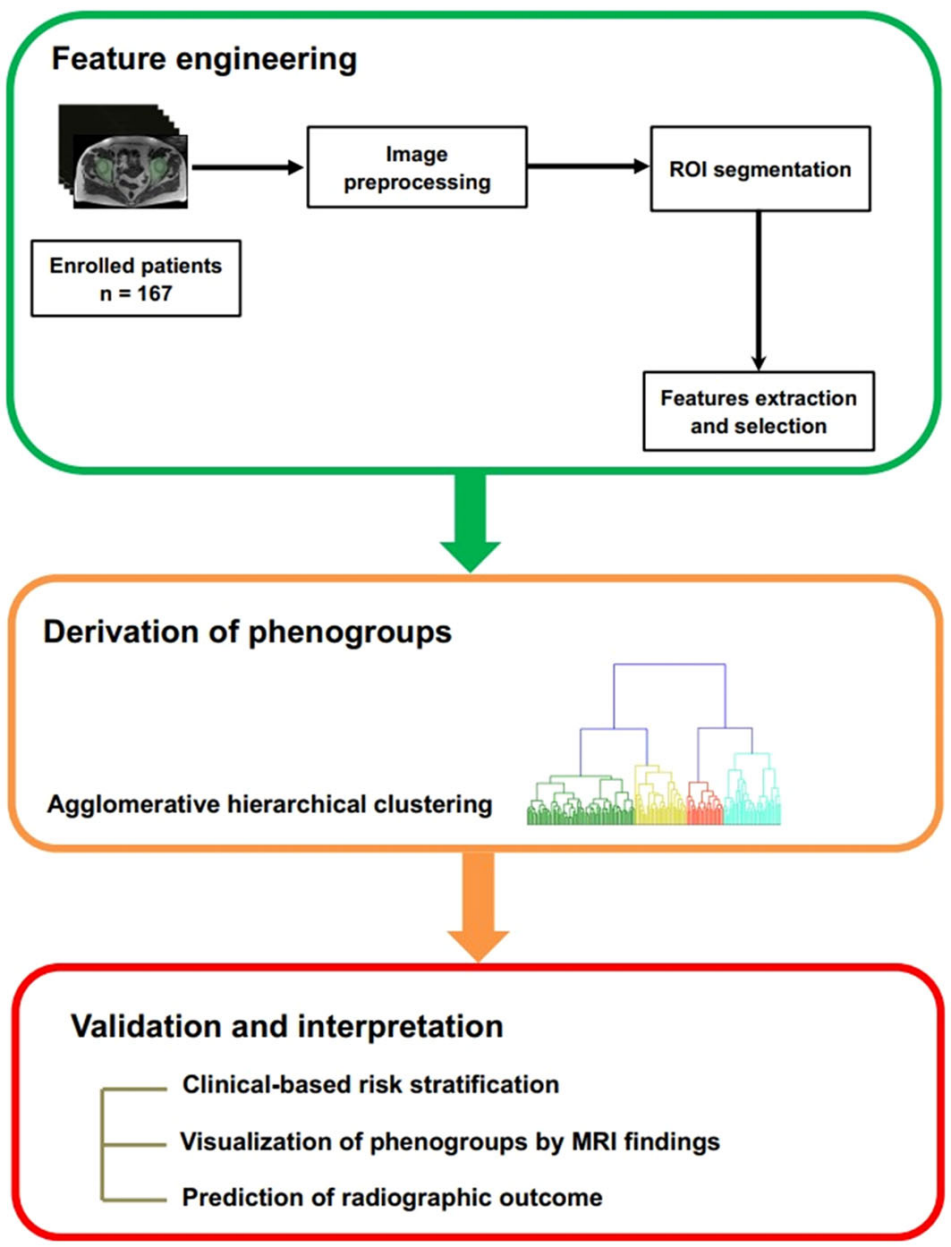
Workflow for the development and validation of the radiomics-based machine learning model. ROI: region of interest.

As the real-world background, patients underwent MRI exams in 8 MRI scanners at our hospital. The parameters of different scanners were detailed in **Supplementary Table S1**. To correct the heterogeneity of radiomic features caused by different scanners, we used a practical realignment approach, the comBat compensation method (17). This method realigns image-derived data in a single space in which the batch effect is discarded. This method enables pooling data from different scanners and centers without a substantial loss of statistical power caused by intra- and inter-center variability (18, 19). Image preprocessing was conducted as a fixed bin size of 25 for image discretization was used to filter noise from images and all images were resampled at the same voxel size (1 × 1 × 1 mm^3^) to standardize the voxel spacing. A detailed workflow of the steps involved in our study was summarized in **Figure 1**.

### Image evaluation and region segmentation

**Figure 2:**
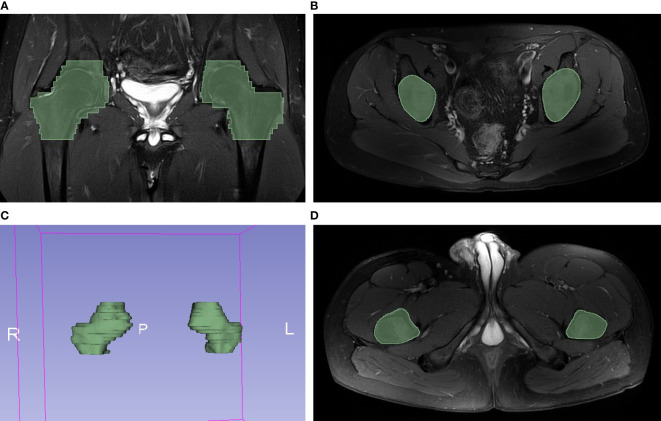
Example of hip MRI slices showed the range of handcrafted segmentation. **(A)** Regions of interest (ROI) of bilateral hips were labeled with green color in coronal plane. **(B)** The first slide containing ROI in axial plane. **(C)** The reconstructed 3D volume of ROI. **(D)** The last slide containing ROI in axial plane.

Conventional MRI characteristics of hip joint were reported by two musculoskeletal radiologists (reader 1 and reader 2). The severity of structure damage of hip joint was also assessed by reader1, according to the BASRI-hip. The presence of joint effusion, BME and enthesitis was considered as active inflammatory changes, whereas sclerosis, subchondral erosion, joint space narrowing and fat lesion were termed as structural damage of hip involvement (7). We defined active inflammatory changes and chronic structural damage with reference to previously reported method (7). Additionally, we used a qualitative method to define these lesions: the presence of a defined lesion in any slice of hip MRI was considered positive for that lesion. A senior radiologist would also be brought into making the final conclusion if there was disagreement between the two observers. Then, a fellowship-trained operator (reader 3) delineated the entire hip joint, composed of the femur, acetabulum, and joint space, as regions of interest (ROI). The reader delineated the ROIs with reference to the range of proximal hip femur, acetabulum and hip joint capsule in slices on an open-source software, 3D Slicer (Version 5.0.3). The ROIs were drawn manually slice by slice in the axial axis, by using edge-based tool and then fine-tuned by the smoothing tool in 3D Slicer (**Figure 2**).

### Radiomic features extraction and selection

Radiomic features were extracted in the open-source radiomics platform, Pyradiomics (version 3.0.1), in Python (version 3.7). Radiomic features were defined according to the Image Biomarkers Standardization Initiative (IBSI) (20) and fell into the following categories: first-order (n=18), shape (n=8) and texture (n=75) features. Moreover, 14 image filters were applied and high-order features (n=1210) were extracted after decompositions of the original images by the filters. A list of all radiomic features and detailed explanation were provided in **Supplementary Table S2**.

Redundancy was checked and radiomic features with invariance were removed. Additionally, to assess the reliability of manual segmentation process, another observer (reader 1) delineated 15 randomly selected patients, after training session and consensus meeting with reader 3. Then, inter-observer (reader 1 and 3) and intra-observer (reader 3 twice) intraclass correlation (ICC) were calculated to evaluate the reliability of extracted radiomic features. Only features with good reproducibility that both inter-observer and intra-observer ICC ≥ 0.75 were considered in further analyses. All selected features were normalized by Z-score standardization before the next step.

### Phenotype derivation, validation and interpretation

Once radiomic features were selected and prepared, unsupervised agglomerative hierarchical clustering with Euclidean distance calculation and Ward linkage criterion was applied to identify radiomics-based patient clusters. Dendrogram that visualizes the clustering procedure and distances between the clusters at different layers was prepared to help determine the optimal number of clusters (phenotypes).

The validation of derived phenotypes was conducted in three ways. First, we characterized the derived phenotypes by clinical backgrounds. In detail, we evaluated inter-groups differences of clinical factors associated with hip involvement, such as juvenile-onset, disease duration, cigarette smoking, TNFi treatment and serum inflammation markers. Second, we interpreted phenotyping results by profiling the heterogeneity of MRI-detected hip lesions between phenotypes. Third, we assessed the radiographic outcomes of hip involvement by the BASRI-hip criteria, to evaluate the performance of radiomics-based phenotyping to predict hip joint structural damage.

### Validation of radiomic-derived phenotypes

To evaluate the robustness and reliability of the phenotypes obtained from unsupervised agglomerative hierarchical clustering, we performed a consensus clustering algorithm using the ‘ConsensusClusterPlus’ package (version 1.62.0). This method involves conducting multiple iterations of clustering on resampled data and then measuring the consistency of the resulting clusters across these iterations (21).

The performance of consensus clustering was assessed using the consensus matrix, cumulative distribution function (CDF) curve, relative alterations in the area under the CDF curve (Delta Area Plot), and cluster-consensus plot, in order to help determine the optimal number of phenotypes and evaluate whether the derived phenotypes are reasonable.

### Statistics

Descriptive statistical analysis was performed using SPSS Statistics (version 22; IBM Corp.). Missing data were addressed using multiple imputation by 5 iterations, assuming they were missing at random. Implementation of other work is based on Python (version 3.7) and R programming language (version 4.2.1). The ICC coefficient was calculated by the two-way mixed effect models and consistency method, by using R package ‘psych’ package (version 2.2.9). Unsupervised agglomerative hierarchical clustering and the formation of dendrogram were based on Python package ‘scikit-learn’ (version 0.22.1). Chord diagrams were created using R package ‘circlize’ (version 0.4.15). We used binary logistic regression to estimate odds ratios (ORs) and 95% CIs of having radiological hip involvement across the derived-phenotypes. For all analyses, two-sided *P* values <0.05 were considered significant.

## Results

### Patients and MRI imaging findings

A total of 167 patients were admitted into our study.146 patients were males (87.4%), the median age (interquartile range (IQR)) was 31.0 (26.0–37.0) years. They had established AS with median disease duration (IQR) of 6 (2.0–10.0) years and their median age (IQR) at disease onset was 23.0 (20.2–28.0). HLA-B27 positive rate was 88.6% and 18 (10.8%) individuals were identified as juvenile-onset AS (JAS). Among the 167 patients, 70 (41.9%) or 71 (42.5%) patients had history of peripheral arthritis or enthesitis, respectively. Besides, 40 (24.0%) patients were ever-smokers and 22 (13.2%) patients had drinking habit.

Joint effusion was the most frequent MRI finding (147, 88.0%), followed by BME (75, 44.9%), erosion (62, 37.1%), fat lesion (59, 35.3%), joint space narrowing (38, 22.8%) and sclerosis (9, 5.4%). Enthesitis was also a prevalent MRI finding and three subtypes were identified based on anatomic location: ischial tuberosity (enthesitis-i, 10 (6.0%)), greater femoral trochanter (enthesitis-t, 61 (36.5%)) and pubic symphysis (enthesitis-p, 34 (20.4%)). Detailed patient characteristics and MRI findings were shown in **Table 1**.

**Table 1:** Characteristics and MRI findings of patients among different phenogroups.

	Total (n= 167)	Phenogroup I (n= 38)	Phenogroup II (n= 34)	Phenogroup III (n= 24)	Phenogroup IV (n= 71)	*P* value
Clinical characteristics						
Age, yrs	31.0 (26.0–37.0)	29.0 (22.0, 33.0)	32.0 (26.0, 37.3)	30.0 (25.3, 35.8)	34.0 (28.0, 37.0)	0.125
Male	146 (87.4%)	30 (78.9%)	28 (82.4%)	24 (100.0%)	64 (90.1%)	**0.046**
JAS	18 (10.8%)	8 (21.1%)	6 (17.6%)	1 (4.2%)	3 (4.2%)	**0.015**
Age at onset, yrs	23.0 (20.2, 28.0)	21.0 (18.5, 24.0)	25.0 (20.8, 28.3)	23.0 (20.2, 28.5)	25.0 (22.0, 30.0)	0.125
Disease duration, yrs	6.0 (2.0, 10.0)	7.0 (3.0, 12.0)	5.0 (2.0, 13.3)	5.0 (3.0, 8.5)	6.0 (2.0, 10.0)	0.840
HLA-B27 (+)	148 (88.6%)	35 (92.1%)	32 (94.1%)	21 (87.5%)	60 (84.5%)	0.483
Peripheral arthritis history	70 (41.9%)	12 (31.6%)	11 (32.4%)	12 (50.0%)	35 (49.3%)	0.165
Enthesitis history	71 (42.5%)	18 (47.4%)	11 (32.4%)	10 (41.7%)	32 (45.1%)	0.579
Smoking status						0.712
None	127 (76.0%)	30 (78.9%)	26 (76.5%)	16 (66.7%)	55 (77.5%)	
Ever smokers	40 (24.0%)	8 (21.1%)	8 (23.5%)	8 (33.3%)	16 (22.5%)	
Alcohol consumption						0.143
None	145 (86.8%)	35 (92.1%)	32 (94.1%)	18 (75.5%)	60 (84.5%)	
With drinking habit	22 (13.2%)	3 (7.9%)	2 (5.9%)	6 (25.0%)	11 (15.5%)	
ESR, mm/h	7.0 (2.0, 18.0)	17.0 (7.0, 49.5)	8.5 (2.0, 19.3)	4.0 (2.0, 11.5)	6.0 (2.0, 13.0)	**< 0.001**
CRP, mg/L	3.4 (1.0, 10.9)	6.5 (2.3, 29.5)	5.6 (1.0, 13.7)	4.1 (1.0, 9.6)	3.0 (0.5, 8.3)	**0.021**
NSAIDs	161 (96.4%)	36 (94.7%)	32 (94.1%)	22 (91.7%)	71 (100.0%)	0.148
TNFi	88 (52.7%)	19 (50.0%)	17 (50.0%)	17 (70.8%)	35 (49.3%)	0.303
TNFi duration, month	4.0 (0.0, 24.0)	30.0 (13.0, 48.0)	20.0 (11.5, 38.0)	20.0 (6.0, 27.0)	21.0 (11.0, 36.0)	0.905
MRI findings						
Joint effusion	147 (88.0%)	36 (94.7%)	29 (85.3%)	20 (83.3%)	67 (94.4%)	0.174
BME	75 (44.9%)	22 (57.9%)	13 (38.2%)	12 (50.0%)	28 (39.4%)	0.230
Enthesitis-t	61 (36.5%)	16 (42.1%)	12 (35.3%)	6 (25.0%)	27 (38.0%)	0.582
Enthesitis-i	10 (6.0%)	6 (15.8%)	0	0	4 (5.6%)	**0.023**
Enthesitis-p	34 (20.4%)	12 (31.6%)	1 (2.9%)	4 (16.7%)	17 (23.9%)	**0.009**
Sclerosis	9 (5.4%)	4 (10.5%)	3 (8.8%)	0	2 (2.8%)	0.169
Erosion	62 (37.1%)	23 (60.5%)	13 (38.2%)	5 (20.8%)	21 (29.6%)	**0.004**
Fat lesion	59 (35.3%)	14 (36.8%)	13 (38.2%)	5 (20.8%)	27 (38.0%)	0.472
Narrowing	38 (22.8%)	17 (44.7%)	7 (20.6%)	2 (8.3%)	12 (16.9%)	**0.002**
Radiological outcomes, (missing = 65)						
BASRI-hip	1.0 (1.0, 3.0)	2.0 (1.0, 4.0)	2.0 (1.0, 3.0)	1.0 (0, 2.0)	1.0 (1.0, 2.0)	**0.027**
Radiological-defined hip involvement	45/102 (44.1%)	16/26 (61.5%)	11/20 (55.0%)	3/13 (23.1%)	13/43 (30.2%)	**0.019**

Data are n (%) for categorical variables and median (interquartile range) for continuous variables, respectively. JAS, juvenile-onset ankylosing spondylitis; ESR, erythrocyte sedimentation rate; CRP, C-reactive protein; NSAIDs, non-steroidal anti-inflammatory drugs; TNFi, tumor necrosis factor inhibitor; BME, bone marrow edema; Enthesitis-t, enthesitis at greater femoral trochanter; Enthesitis-i, enthesitis at ischial tuberosity; Enthesitis-p, enthesitis at pubic symphysis; BASRI-hip, Bath ankylosing spondylitis radiology hip index. Bold text highlighted significant differences.

### Radiomic features and phenotypes derivation

**Figure 3:**
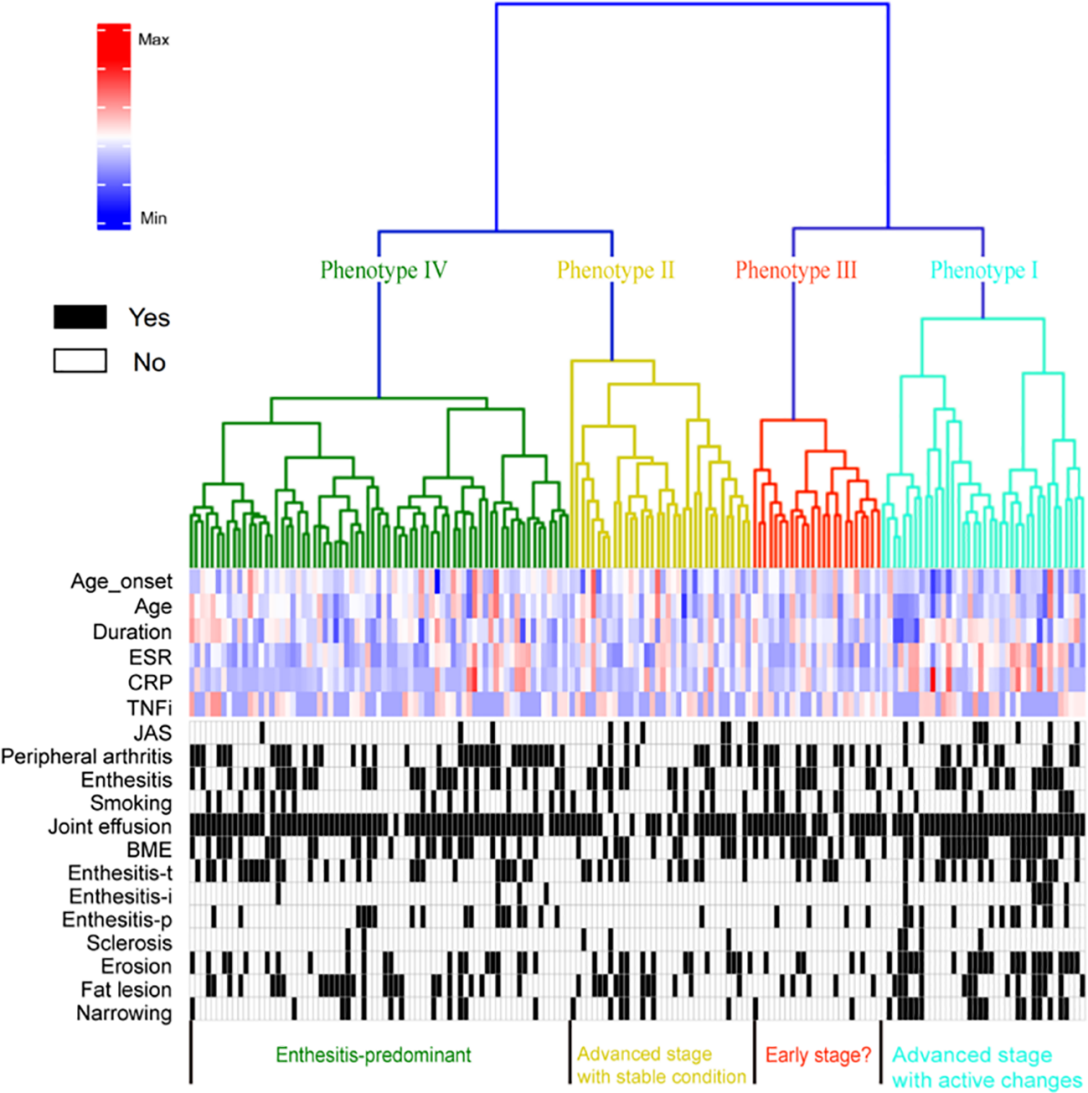
Dendrogram shows the process of unsupervised hierarchical clustering. Heatmap shows results of the cluster analysis of patient clinical profiles and MRI-detected lesions. ESR, erythrocyte sedimentation rate; CRP, C-reactive protein; TNFi, tumor necrosis factor inhibitor; JAS, juvenile-onset ankylosing spondylitis; Peri_history, Peripheral arthritis history; E_history, Enthesitis history; BME, bone marrow edema; Enthesitis-t, enthesitis at greater femoral trochanter; Enthesitis-i, enthesitis at ischial tuberosity; Enthesitis-p, enthesitis at pubic symphysis.

1422 radiomic features were extracted based on T2WI MRI images. After removing redundant and instable features, 1321 robust radiomic features were identified and used for model construction. The agglomerative hierarchical clustering model identified four phenotypes of patients (**Figure 3**). Characteristics including demographics, clinical variables, serum inflammation markers and previous treatments across the four phenotypes were presented in **Table 1**.

Phenotype I consisted of 38 (22.8%) patients. Compared to the others, it included more younger (median age 29.0 years, IQR (22.0, 33.0)) and JAS (8, 21.1%) patients. Besides, patients in phenotype I had longer AS duration (7.0 (3.0, 12.0)) and significantly elevated serum inflammatory markers (17.0 (7.0, 49.5) and 6.5 (2.3, 29.5) for ESR and CRP, respectively). Phenotype II consisted of 34 (20.4%) patients. As similar to phenotypes I, phenotypes II included patients with high rate of juvenile-onset (6, 17.6%) and elevated serum inflammatory markers (8.5 (2.0, 19.3) and 5.6 (1.0, 13.7) for ESR and CRP, respectively). The TNFi use rate in phenotypes II was similar to that in phenotype I (50.0% vs 50.0%, *P*=0.593) but phenotypes II had shorter duration of TNFi use than phenotypes I (20.0 (11.5, 38.0) vs 30.0 (13.0, 48.0), *P*=0.043).

Phenotype III consisted of 24 (14.4%) patients and phenotype IV included 71 (42.5%) patients. They shared similar characteristics that patients were neither apt to be JAS (4.2% and 4.2% for phenotype III and IV, respectively) nor had elevated serum inflammatory markers (ESR 4.0 (2.0, 11.5) and 6.0 (2.0, 13.0), CRP 4.1 (1.0, 9.6) and 3.0 (0.5, 8.3) for phenotype III and IV, respectively). As for TNFi treatment, the duration of TNFi use in phenotype III (20.0 (6.0, 27.0)) and IV (21.0 (11.0, 36.0)) were comparable to phenotype II (20.0 (11.5, 38.0), despite more frequent TNFi use in phenotype III (50.0%, 70.8% and 49.3% for phenotype II, III and IV, respectively, *P*= 0.905).

Therefore, according to their exposure on known clinical factors associated with hip involvement, phenotype I and II could be labelled as high-risk while phenotype III and IV were at low-risk for hip involvement in AS.

### Validation of radiomic-derived phenotypes by consensus clustering

**Figure 4:**
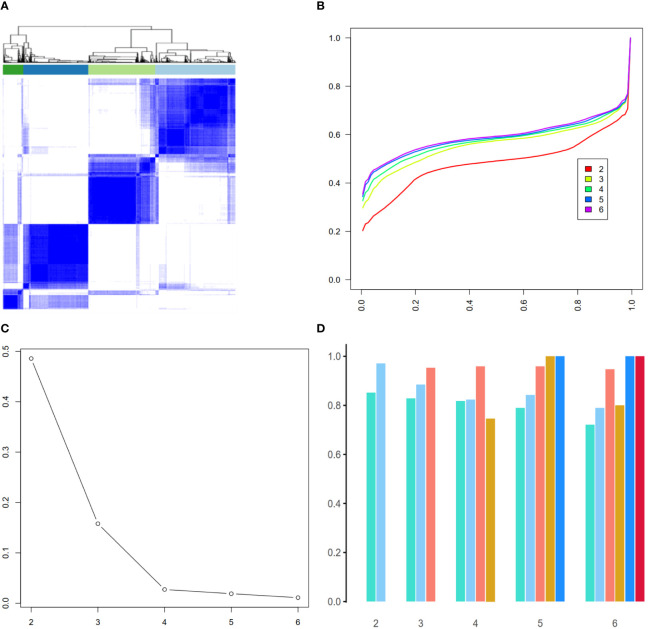
Validation of radiomic-derived phenotypes by consensus clustering. **(A)**: Consensus matrix when *k* = 4. **(B)** Consensus CDF curves when *k*=2 to 6. **(C)** Relative alterations in CDF Delta area plot. **(D)** Cluster-consensus value of each phenotype when *k*=2 to 6.

To assess the robustness of the derived 4-phenotype structure of radiomics data, we performed consensus clustering to validate the radiomics-based phenotypes. Based on the consensus matrix (**Figure 4A**), CDF curve (**Figure 4B**), Delta area plot (**Figure 4C**), *k* = 4 was identified as the optimal value for phenotyping the AS patients. Additionally, as expected, these four phenotypes had high cluster-consensus values (**Figure 4D**), indicating strong stability among the radiomic-derived phenotypes.

### Interpretation of four phenotypes by MRI findings

Both phenotype I and II manifested high prevalence of structural lesion. More specifically, the high-risk phenotypes were associated with significantly higher prevalence of erosive lesion [36 (50.0%) vs 26 (27.4%), odds ratio (OR) 2.65 (95% CI 1.39, 5.06)] and joint space narrowing [24 (33.3%) vs 14 (14.7%), OR 2.89 (95% CI 1.37, 6.12)] than the low-risk, whereas they did not differ for sclerosis and fat lesion. In contrast, phenotype II had lower prevalence of active lesions than phenotype I (joint effusion (85.3% vs 94.7%, *P*=0.243), BME (38.2% vs 57.9%, *P*=0.096), enthesitis-t (35.3% vs 42.1%, *P*=0.554), enthesitis-i (0 vs 15.8%, *P*=0.026) and enthesitis-p (2.9% vs 31.6%, *P*=0.002)), which reflected that phenotype II had severe structural damage but less active inflammatory lesions on MRI.

**Figure 5:**
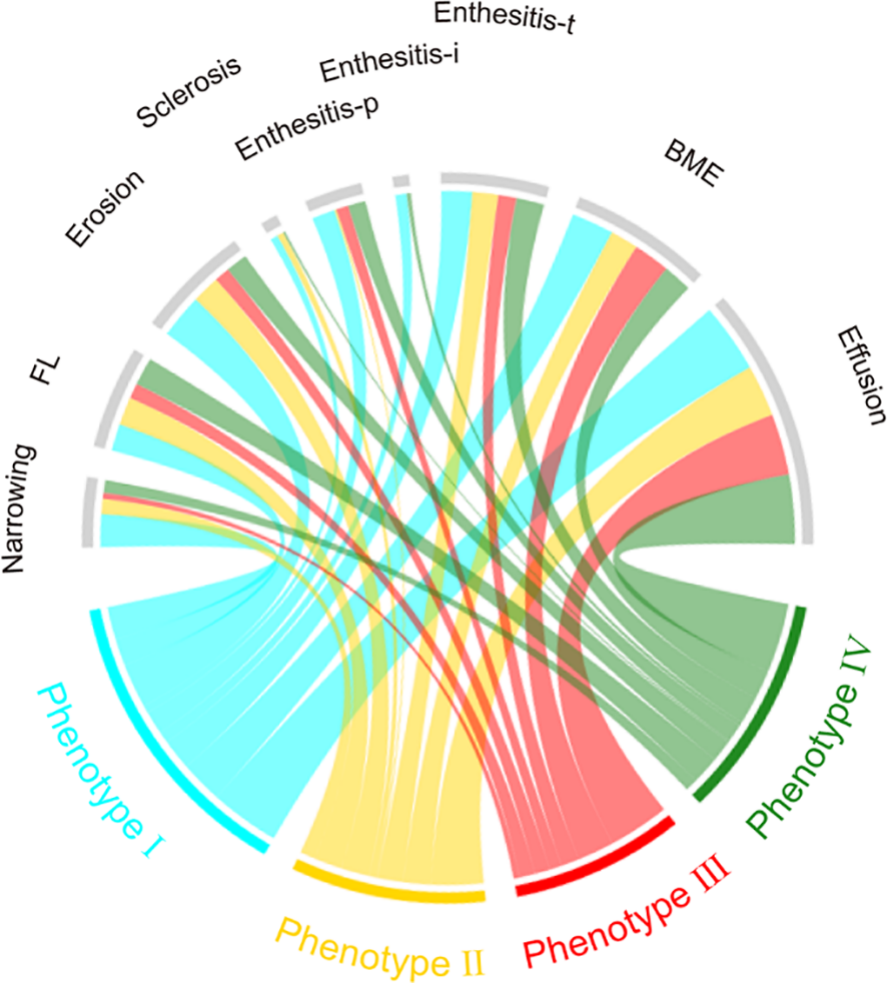
Chord diagrams showing differences in MRI findings among phenotypes. BME, bone marrow edema; Enthesitis-t, enthesitis at greater femoral trochanter; Enthesitis-i, enthesitis at ischial tuberosity; Enthesitis-p, enthesitis at pubic symphysis; FL, Fat lesion.

As for acute inflammatory signs, the high-risk phenotypes had comparable prevalence of joint effusion [65 (90.3%) vs 87 (91.6%), OR 0.46 (95% CI 0.18, 1.19)], BME [35 (48.6%) vs 40 (42.1%), OR 1.30 (95% CI 0.70, 2.41)] and enthesitis-t [28 (38.9%) vs 33 (34.7%), OR 1.20 (95% CI 0.63, 2.26)] than the low-risk phenotypes. Nevertheless, phenotype I and IV had significantly higher prevalence of enthesitis-i (15.8% and 5.6%, respectively, *P*=0.023) and enthesitis-p (31.6% and 23.9%, respectively, *P*=0.009) compared to phenotype II and phenotype III (enthesitis-i: 0 for both, enthesitis-p: 2.9% and 16.7%, respectively). MRI findings across the 4 phenotypes were presented in **Table 1** and inter-group differences were visualized in **Figures 3**, **5**.

### Prediction of radiographic outcomes by phenotypes

102 patients received pelvis X-ray exams at a 2-year interval after taking MRI exams. Patients in phenotype I and II had significantly higher BASRI-hip scores than phenotype III and IV (median (IQR) of scores were 2.0 (1.0, 4.0), 2.0 (1.0, 3.0), 1.0 (0, 2.0) and 1.0 (1.0,2.0), respectively, *P*=0.027). Likewise, after adjusting for confounding factors including JAS, age, duration, smoking status and ESR, the high-risk phenotypes (phenotype I and II) were 3 times more likely to have radiological-defined hip involvement (BASRI-hip ≥ 2) than the low-risk [27 (58.7%) vs 16 (28.6%), adjusted OR 2.95 (95% CI 1.10, 7.92)].

Therefore, according to clinical behaviors, MRI characteristics and radiographic outcomes, patients in phenotype I and II could be labeled as “advanced-stage hip involvement”. Patients in phenotype I concomitantly exhibited significant acute inflammation signs and demanded anti-inflammatory therapy, especially TNFi treatment. Phenotype III and IV were assumed as “early-stage hip involvement”, and phenotype IV was enthesitis-predominant, whereas patients in phenotype III were not yet identified based on the current variables.

## Discussion

Hip involvement is prevalent in AS and constitutes an important reason of disability in AS (2, 3). There remains unmet need that a method can make early and accurate identification of hip involvement in AS, as early detection means the opportunity to get timely treatments. Radiomics has gained increasing attention in the last few years, as a promising quantitative image analyzing method used for differential diagnosis, prognosis analysis and identification of responders to therapy (22, 23). In this pilot study, four distinct phenotypes of AS-related hip involvement were identified by the integration of MRI radiomics data and unsupervised ML approach. This study is, to the best of our knowledge, the first to apply radiomics-based approach to profile hip involvement in AS. Our study validated the clinical actionability of using radiomics approach to detect hip involvement in AS, which offers opportunities for the foundation of a novel method, the MRI radiomics, to diagnose hip involvement in AS.

A 4-phenotype structure of radiomics data were derived and it was validated from the perspectives of clinical backgrounds, MRI signs and radiographic outcomes. Firstly, phenotype I and II were labelled as high-risk clinical pattern, in that they included more patients exposed to risk factors associated with hip involvement than the other two phenotypes (low-risk clinical pattern). Then, we used conventional MRI findings to validate the phenotyping structure and interpreted the radiomics-based phenotypes, since the ‘black-box’ nature of artificial intelligence-based approaches often provides results that are difficult to understand (24). Practitioners are more familiar with the clinical implications of MRI findings rather than radiomic features. Importantly, the significantly increased prevalence of MRI-detected structural damage on high-risk than low-risk phenotypes vigorously supported such clinical patterns. Additionally, patients in phenotype I had notable acute inflammation signs besides the presence of structural damage while phenotype IV was assumed as “enthesitis-predominant”, given the prominent enthesitis findings on MRI. The profiling of phenotype III was challenging since it had limited cases number (only 24 patients). Patients in phenotype III were young and less likely exposed to risk factors associated with hip involvement, we carefully inferred that their nonspecific MRI findings may derive from other origins of hip joint pain, such as stress injury, acute bone marrow edema syndrome or femoroacetabular impingement (25, 26), besides the possibility that they represent a stage, probably the early stage, in the progression of AS-related hip involvement.

The radiographic outcomes of hip involvement strongly supported the current phenotyping results. After adjusting for confounding factors, patients with high-risk phenotypes were associated with 3.0-fold higher odds of having radiological hip involvement than the low-risk (ORa 2.95 (95% CI 1.10, 7.92)). This finding suggested that radiomics-derived phenotyping could predict the radiographic outcome of hip involvement in AS, which makes the radiomics method a promising tool in the early identification of hip involvement in AS. Additionally, consensus clustering analysis significantly enhances the credibility and robustness of our findings. These results endorse that the derived phenotypes are not only statistically sound but also clinically interpretable and meaningful.

Among the reported MRI findings associated with hip involvement in AS, we don’t know which were of predictive power for worse outcome or which could discriminate it from other reasons of hip pain. Our study provided some indirective evidence for this question. Joint effusion is an indirective MRI finding of hip synovitis and BME is linked to bone marrow capillary wall damage and leakage (5). Joint effusion and BME were quite common MR findings in AS patients with hip joint pain (7) but they had a low-level variance among the 4 phenotypes. Erosion, sclerosis and joint space narrowing were structural lesion findings in MRI, their roles were quite limited since the target was early diagnosis of hip involvement. Focal fat infiltration likely reflects post-inflammatory tissue metaplasia: since the inflammation recedes, fat metaplasia develops in its place (27, 28). The prevalence of fatty lesion was comparable in phenotype I, II and IV (36.8%, 38.2% and 38.0%, respectively), despite it subtle decreased in phenotype III (20.8%). We also found that enthesitis was a prevalent MRI finding in each phenotype and it comprised one distinct phenotype of patients. Further studies are needed to dissect the pathophysiologic significance of fat lesion and enthesitis in hip joints and their value in sorting out AS-related hip involvement from other origins of hip joint pain. It is noteworthy that we evaluated the described MRI signs in a crude mode that whether they existed or not and the emergence of sophisticated methods such as morphological feature analysis, quantitative scoring and radiomic feature analysis, had shed light on exploring of AS-specific MRI findings (10, 29, 30).

Our study has several limitations that should be acknowledged. Firstly, there existed sampling bias due to various factors, including relatively young population and a geographical area where AS population had limited biologics use (31), which may render a relative high prevalence of hip involvement. Additionally, we enrolled patients with AS (radiographic axial SpA) rather than non-radiographic axial SpA, which was assumed as the pre-stage of axial SpA (1). Further researches are needed to investigate whether our observations persist across racial, ethnic and the whole SpA groups. Secondly, we did not set out a specific prediction model or scoring system for the prediction of hip involvement in AS, which we believe requires further developed tools as well as external validation. Rather, we aimed to ascertain the potential of MRI radiomics approach to profile hip involvement in AS. We believed that the novelty predominantly lies in the described methodology, and perhaps less so in the detected four phenotypes, despite that they were comprehensively validated. Finally, patients in phenotype III were not yet identified and the underlying cellular or molecular level heterogeneity across the four phenotypes were not studied.

In conclusion, our results serve as a proof-of-concept that unsupervised ML methods could turn complex radiomics data into interpretable and clinically meaningful classification of hip involvement in AS. Our findings illuminate a promising approach to identify hip involvement in AS and its added value in clinical decision making should be evaluated in prospective studies.

## Data Availability

The original contributions presented in the study are included in the article/**Supplementary Material**. Further inquiries can be directed to the corresponding author.
