# Supplementary material for: Radiomics-based machine learning model to phenotype hip involvement in ankylosing spondylitis: a pilot study
Source: Front Immunol. 2024 Aug 29;15:1413560. doi: 10.3389/fimmu.2024.1413560 (PMC11390496; doi:10.3389/fimmu.2024.1413560)
Supplement: Supplementary file 1 [file Table1.docx]

|  | Slice thickness, mm | Interval, mm | Repetition time, ms, | Echo time, ms | FOV,  mm*mm |
| --- | --- | --- | --- | --- | --- |
| Scanner 1 |  |  |  |  |  |
| Siemens, Aera 1.5T | | | | | |
| T1WI | 4 | 1 | 620 | 7.7 | 360*360 |
| T2WI | 4 | 1 | 4100 | 60 | 360*360 |
| Scanner 2 |  |  |  |  |  |
| Philips, Ingenia 3.0 T | | | | | |
| T1WI | 4 | 0.4 | 670 | 20 | 300*381 |
| T2WI | 4 | 0.4 | 2848 | 60 | 300*378 |
| Scanner 3 | | | | | |
| Alltech, EchoStar 1.5T | | | | | |
| T1WI | 4 | 0.8 | 600 | 13.6 | 380*380 |
| T2WI | 4 | 0.8 | 4000 | 47.5 | 380*380 |
| Scanner 4 | | | | | |
| United Imaging, uMR 770 3.0 T | | | | | |
| T1WI | 4 | 0.4 | 725 | 10.6 | 380*380 |
| T2WI | 4 | 0.4 | 3476 | 30.9 | 380*380 |
| Scanner 5 |  |  |  |  |  |
| GE Healthcare, Discovery MR750w 3.0T | | | | | |
| T1WI | 4 | 1 | 472 | 11.6 | 380*380 |
| T2WI | 4 | 1 | 4606 | 63.2 | 380*380 |
| Scanner 6 |  |  |  |  |  |
| Siemens, Skyra 3.0T | | | | | |
| T1WI | 4 | 1.2 | 795 | 23 | 380*380 |
| T2WI | 4 | 1.2 | 4000 | 39 | 380*380 |
| Scanner 7 |  |  |  |  |  |
| United Imaging, uMR570 1.5T | | | | | |
| T1WI | 4 | 0.4 | 549 | 13 | 380*380 |
| T2WI | 4 | 0.4 | 3031 | 28 | 380*380 |
| Scanner 8 |  |  |  |  |  |
| United Imaging, uMR560 1.5T | | | | | |
| T1WI | 4 | 0.8 | 600 | 13.6 | 350*350 |
| T2WI | 4 | 0.8 | 4000 | 47.5 | 350*350 |

*Supplementary Table 1: The Parameters of different MRI scanners in this study.*
